# New Biomarkers for Systemic Necrotizing Vasculitides

**DOI:** 10.3390/jcm13082264

**Published:** 2024-04-13

**Authors:** Russka Shumnalieva, Plamena Ermencheva, Georgi Kotov, Iva Parvova-Hristova, Konstantina Bakopoulou, Issa El Kaouri, Niya Mileva, Tsvetelina Velikova

**Affiliations:** 1 Department of Rheumatology, Clinic of Rheumatology, University Hospital St. Ivan Rilski, Medical University of Sofia, 13 Urvich St., 1612 Sofia, Bulgaria; rshumnalieva@yahoo.com (R.S.); p.ermencheva@gmail.com (P.E.); gn_kotov@abv.bg (G.K.); ivaparvova@mail.bg (I.P.-H.); 2 Medical Faculty, Sofia University, St. Kliment Ohridski, 1 Kozyak Str., 1407 Sofia, Bulgaria; tsvelikova@medfac.mu-sofia.bg; 3 Faculty of Medicine, Medical University of Sofia, 1 Georgi Sofiiski Str., 1431 Sofia, Bulgaria; 104170@students.mu-sofia.bg (K.B.); 104166@students.mu-sofia.bg (I.E.K.)

**Keywords:** biomarkers, systemic necrotizing vasculitides, diagnostic, prognostic, inflammatory markers, precision medicine, immunological indicators, disease monitoring

## Abstract

Systemic necrotising vasculitides (SNVs) pose significant challenges due to their diverse clinical manifestations and variable outcomes. Therefore, identifying reliable biomarkers holds promise for improving precision medicine in SNVs. This review explores emerging biomarkers aiming to enhance diagnostic accuracy, prognostic assessment, and disease monitoring. We discuss recent advances in immunological biomarkers, inflammatory indicators, and other parameters that exhibit potential diagnostic and prognostic utility. A comprehensive understanding of these biomarkers may facilitate earlier and more accurate SNV detection, aiding in timely intervention and personalized treatment strategies. Furthermore, we highlight the evolving landscape of disease monitoring through innovative biomarkers, shedding light on their dynamic roles in reflecting disease activity and treatment response. Integrating these novel biomarkers into clinical practice can revolutionize the management of SNVs, ultimately improving patient outcomes and quality of life.

## 1. Introduction

Systemic necrotizing vasculitides (SNVs) refer to a group of diseases characterized by blood vessel inflammation associated with vessel wall necrosis. This leads to subsequent vessel occlusion, vasoconstriction and finally, end-organ damage [1]. Most SNVs are marked by multiorgan involvement and long-term morbidity. Common, potentially life-threatening complications include stroke, glomerulonephritis, bowel infarction and heart failure. Immune mechanisms related to their pathogenesis include deposition of immune complexes, presence of antibodies—commonly, against neutrophilic proteins—antineutrophil cytoplasmic antibodies (ANCAs) and cell-mediated tissue inflammation [1,2].

The classification of SNVs includes Polyarteritis Nodosa (PAN, Kawasaki disease (KD), ANCA-associated vasculitides (AAVs), IgA vasculitis (IgAV), cryoglobulinemic vasculitis (CV) and Goodpasture’s syndrome (GS) [3,4]. The group of AAV includes granulomatosis with polyangiitis (GPA), microscopic polyangiitis (MPA), and eosinophilic granulomatosis with polyangiitis (EGPA) [5].

Although SNV is diagnosed based on a constellation of clinical features, combined with a biopsy of involved tissue or angiography to demonstrate the characteristic vascular pathology, early diagnosis is challenging [6]. Furthermore, the non-specific clinical manifestations can mimic several infectious, neoplastic, and autoimmune disorders. The wide differential diagnosis, the invasiveness of the procedures, the demand for specific diagnostic resources, and the time-consuming nature of the diagnosis of SNV pose significant issues.

The group incidence of SNVs is relatively low. Still, they pose a severe life-threatening risk, with a combined mortality rate almost reaching one-fifth of cases (19.8%), according to Pagnoux et al. [4]. Therefore, high mortality makes SNV a condition requiring timely diagnosis and appropriate treatment so that remission is achieved, and lethality is prevented. Consequently, it is essential to differentiate the manifestations of active disease in need of active immunosuppressive intervention from simple manifestations of the disease [2].

Biomarkers, as their name “biological markers” shows, are any substance or its products that indicate a biological or a pathogenic state at a given moment and can be objectively measured and evaluated. Biomarkers are used in clinical practice for the assessment of disease activity, outcome prediction or effect of a therapeutic intervention [7].

An ideal biomarker should be both sensitive and specific to the disease. The variety of presently used biomarkers remains very limited, with multiple biomarkers serving more than one diagnostic entity. For instance, ANCA can signify infections, inflammatory bowel disease, or drug-induced vasculitis, whereas a positive cryoglobulin test may indicate CV, as well as hepatitis C or human immunodeficiency virus (HIV), leading to acquired immunodeficiency syndrome (AIDS) [8]. Therefore, currently available biomarkers lack sufficient sensitivity and specificity to be used independently and require additional tests. This knowledge gap is currently the subject of numerous studies in progress. 

This review outlines the diagnostic challenges surrounding SNV, the role of current and potential new biomarkers in its diagnosis and disease assessment, the current areas of research interest, and their implications in clinical practice.

## 2. Current Challenges in SNV Diagnosis and Management

### 2.1. Overview of SNV Clinical Presentation

Purpura, fever, arthralgia, hemoptysis, pulmonary hemorrhage, and abdominal pain encompass the wide range of presenting manifestations of SNV, as highlighted by Younger et al. [9]. Additionally, the five primary clinical cutaneous presentations include palpable purpura, urticarial, infarction-ulcerative, nodular, and livedoid lesions; they appear most frequently in the lower extremities [10].

However, SNV sometimes exhibits uncharacteristic clinical signs, thereby rendering the diagnostic process difficult. Given the rarity of SNV, these infrequent symptoms further complicate the challenging diagnosis as noted by Sharma et al. [11].

Sharma et al. presented a study involving five case studies outlining the various atypical forms SNV may present clinically. These ranged from a subcutaneous abscess in the thigh to advanced renal failure and a combination of PAN with antiphospholipid syndrome and Budd–Chiari syndrome [11]. Considering these unusual presentations, we can conclude that the diagnostic approach of SNV is demanding and should be met with a high degree of clinical suspicion.

### 2.2. Limitations of Existing Diagnostic Tools

Considering that the initial signs of SNV are non-specific, a combination of these symptoms is required to form a diagnosis [12]. Furthermore, there is a wide spectrum of medical conditions that resemble the clinical manifestations of vasculitis, consequently leading to various differential diagnoses. Additionally, along with occurring as a primary disorder, SNV may be secondary to underlying conditions [13]. In line with this, the diagnostic process depends on many criteria that hinder a definite diagnosis and bank upon a biopsy of an impaired organ. A biopsy is considered the gold standard [12]. Biopsy findings may not be valuable as histological examination could be unremarkable or demonstrate non-specific results. Furthermore, obtaining a histological sample may be difficult, in which case an angiogram is considered [13]. Reliance on biopsy results may delay the initiation of treatment, which may prove to be risky. Moreover, the necessity for a specific and accurate diagnosis is further exemplified due to varying treatment plans depending on the various forms of vasculitis, as some forms may be life-threatening [13].

### 2.3. Importance of Early Detection and Prognostic Evaluation

Several risk factors are associated with a greater mortality risk, as noted by Guillevin et al. These include age >65, renal insufficiency, cardiac insufficiency, and gastrointestinal involvement [14]. As further evidenced by Jarder et al., advanced age at diagnosis of patients with MPA is in part linked to increased mortality rates [15]. In patients without a diagnosis and treatment, mortality rates were 100% in a study conducted by Terrier et al., leading to the conclusion that early detection of SNV is associated with improved survival [10]. On the other hand, ear, nose and throat (ENT) manifestations of SNV are attributed to an improved outlook in GPA and EGPA vasculitis. Kaplan–Meier analysis showed comparable mortality rates over 5 years; for patients with GPA vasculitis, the mortality rate was 23% for those with ENT manifestations and 43% without, thus emphasizing the importance of adequate prognostic evaluation [15]. Due to improvements in the early detection of cardiovascular disease associated with SNV, mortality rates have significantly decreased, as presented by Jarder et al. [15]. This highlights the importance of early detection and the evaluation of prognostic markers to better form a treatment plan.

## 3. Role of Biomarkers in SNV

In line with this, new advances in the pathogenesis and treatment strategies in SNV raise the need for new potential biomarkers that better correlate with the disease activity and predict the risk of relapses or treatment response.

### 3.1. Proinflammatory Biomarkers

Traditional non-specific markers of inflammation include erythrocyte sedimentation rate (ESR) or C-reactive protein (CRP), which correlate with the activity of the disease. Increased eosinophil count is the hallmark of EGPA, but it responds rapidly to treatment with glucocorticoids and thus is not a promising biomarker for disease activity [16,17]. Calprotectin is a potential disease biomarker in patients with AAV by showing that patients with AAV had higher monocyte and neutrophil cell surface calprotectin expression than healthy controls (HCs) and that its levels increased following treatment withdrawal and were significantly elevated in patients who relapsed [18].

### 3.2. Autoantibodies

The correlation of several autoantibodies with the disease entity, activity, monitoring and progression serves as a valuable diagnostic and prognostic tool in clinical practice. ANCAs are widely used as diagnostic biomarkers in SNV affecting predominantly small vessels, including GPA, MPA, EGPA and their localized forms (e.g., pauci-immune necrotizing and crescentic glomerulonephritis) combined under the term AAV [17]. The two main patterns of ANCAs on indirect immunofluorescence (IIF) are diffuse granular cytoplasmic staining with interlobular accentuation (cANCA) and perinuclear fluorescence with nuclear extension (pANCA) pattern where the main target of cANCA is proteinase 3 (PR3) and the main target of pANCA is myeloperoxidase (MPO) detected by enzyme-linked immunosorbent assay (ELISA). The atypical ANCA pattern (A-ANCA) has been described in other autoimmune diseases, as well as in drug-induced AAV, and its role has not yet been revealed [19]. The recognition of the two main target autoantigens of ANCA, MPO and PR3, identified ANCA-positive disease subtypes but with distinctions in clinical phenotype, genetic basis, histological findings, epidemiology and response to therapy [20]. As mentioned, IIF of ethanol-fixated neutrophils and ELISA for antigen specificity are the main assays for detecting ANCAs. IIF subdivides ANCA into two major staining patterns: p-ANCAs, mainly composed of MPO, and c-ANCA, of which the most important is PR3. Other ANCAs have also been described, including those against α-enolase, bactericidal permeability-increasing protein (BPI), cathepsin G, elastase, and lactoferrin, but they are rarely associated with vasculitis [21]. ANCA serotyping distinguishes different classes of AAVs: PR3-ANCA AAV, MPO-ANCA AAV, and ANCA-negative AAV [22].

A lesser-known antigen target of ANCA is the lysosome-associated membrane protein-2 (LAMP-2), which, in contrast to PR3 and MPO, is expressed on many cell types, including endothelial cells and, in particular, on the surface of the renal microvascular endothelium. Autoantibodies against LAMP-2 are suggested to play a role in the pathogenesis of ANCA-negative pauci-immune necrotizing glomerulonephritis [23]. Moreover, serum levels of LAMP-2 are found to reflect the disease activity and renal involvement of small and medium vessel vasculitis (SMVV) and were reported to be significantly higher in PAN and cPAN compared to AAV [24,25]. Cryoglobulins are immunoglobulins that precipitate or form a gel in vitro at a temperature less than 37 °C. The presence of monoclonal immunoglobulins defines cryoglobulinemia type I, the presence of polyclonal immunoglobulin with monoclonal rheumatoid factor IgM defines cryoglobulinemia type II, and the presence of polyclonal IgM and IgG defines cryoglobulinemia type III [26]. Cryoglobulinemic vasculitis (CV) usually manifests in type II or type III cryoglobulinemia and is mainly associated with hepatitis C infection. The pathogenic mechanism behind CV is a complex-mediated vessel wall inflammation [26,27]. Anti-glomerular basement membrane (anti-GBM) antibodies have a diagnostic capacity in anti-GBM disease, formerly known as Goodpasture’s syndrome (GS). The disease is thought to be auto-antibody mediated and presents with pulmonary and renal involvement. Crescentic glomerulonephritis causes rapidly progressive glomerulonephritis due to linear deposition of anti-GBM-Ab type IgG on glomerular capillaries. The disease progression correlates with the titer of anti-GBM antibodies, and the latter could also be used for disease monitoring [3,28].

Other autoantibodies of interest are anti-endothelial cell antibodies (AECAs), a heterogeneous family of antibodies that specifically recognize proteins and molecules on the endothelial cell’s surface. AECAs have been found in various systemic diseases, including primary vasculitides. The suggested pathogenic mechanism in the vasculitic lesions is the upregulation of adhesion molecules and expression of cytokines and chemokines by AECAs. AECA levels fluctuate with disease activity, but their clinical significance needs to be determined [29,30].

### 3.3. Endothelial Damage Biomarkers

Two important biomarkers of endothelial injury were studied in AAV: endothelial microparticles (EMPs) and circulating detached mature endothelial cells (CECs). Research on EMPs identified a positive correlation with disease activity compared to patients in remission [20].

### 3.4. Immunoglobulins and Complements

In patients with IgA vasculitis (IgAV), increased serum levels of poorly galactosylated IgA1 remain the most consistent finding in patients with IgA nephritis and IgA nephropathy [7]. Renal biopsy is essential for diagnosing IgAV-N, probably guiding treatment and predicting outcome; the procedure cannot be used repeatedly during patient follow-up [31]. In patients with active AAV, there were significantly higher urinary levels of Bb, C3a, C5a, and soluble C5b-9 [20].

### 3.5. B Cells, T Cells, Cytokines, Chemokines—Serum and Urine

Altered expression of matrix metalloproteinases (MMPs) and tissue-inhibitors of matrix metalloproteinases (TIMPs) are important molecules in the pathogenesis of vessel wall inflammation and injury as well as in vascular repairment. MMPs and TIMPs have been found in the circulation, urine, and kidney and lung tissues in patients with AAV. Levels of MMP-2, MMP-9 and TIMP-1 were elevated in the urine as a possible biomarker for ANCA-associated kidney involvement, and circulation levels of MMP-2, MMP-7 and TIMP-2 were found to better discriminate active disease from remission. MMP-2, MMP-7 and TIMP-2 were the best potential discriminators between active disease and remission. MMP-3, MMP-7, and TIMP-1 were related to renal function in these patients. MMP-9 and TIMP-1 showed a correlation with disease activity in AAV, and TIMP-1 was the best discriminator between mildly active AAV and remission [32,33]. Moreover, MMP-9, known to control monocyte and T-lymphocyte invasion of the vascular wall, together with macrophage migration inhibitory factor (MIF) are found to be increased in both PR3-ANCA and MPO-ANCA vasculitide. Currently, there is no routine clinical utility of MMPs or TIMPs in SNVs [34,35].

In several studies, monocyte chemotactic protein 1 (MCP-1) was the most promising urinary biomarker for AAV, suggesting its usefulness in disease assessment and treatment monitoring [36,37,38]. Urinary soluble CD163 (sCD163) levels were found to be higher in patients with active AAV compared to those in remission [39]. However, serum sCD163 levels failed to distinguish infections from active disease, which may limit its use [40]. A multicenter study showed that urinary CD89 and transglutaminase2 (TG2) concentrations are significantly lower in patients with active IgA vasculitis and nephritis compared to individuals whose disease has gone into complete or partial remission [31]. Incomplete B-cell depletion and repopulation after treatment with rituximab have been associated with a significantly higher rate of relapses in AAV patients [41]. Moreover, Xu et al. reported disrupted humoral immune responses in AAV patients caused by the imbalance between circulating T follicular helper (Tfh) cells and T follicular regulatory (Tfr) cells. There is an elevated Tfg/Tfr ratio compared with HCs and a Tfh2/ Tfh1 shift with an increased plasma level of interleukin (IL)-21, which was found to be associated with AAV and disease activity [42].

### 3.6. Genetic and Epigenetic Biomarkers

The autoimmune character of SNV suggests the possible role of genetic and epigenetic mechanisms in disease pathogenesis. Genome-wide association studies (GWASs) have highlighted that MHC class II polymorphisms may influence the development of particular ANCA serotypes but not the clinical phenotype of AAV [7]. Genetic polymorphisms of six HLA class I genes have been linked with KD alongside MICA alleles A4 and A5.1. ESR and N-terminal pro-b-type natriuretic peptides (NT-proBNP) are useful and promising as components of a diagnostic toolkit for KD [43]. The detection of circulating free DNA (cfDNA) levels or NETs may serve as a marker of disease activity in AAV, namely PR3-ANCA-positive [44]. MicroRNAs (miRNAs) are a class of endogenous short noncoding RNA molecules that negatively regulate the gene expression at the posttranscriptional level by targeting messenger RNAs (mRNAs) [45,46]. Besides influencing the pathogenesis of KD, miRNA is an extraordinary biomarker for diagnosing and classifying KD patients [47]. So far, the detection of single nucleotide polymorphisms (SNPs) has limited clinical utility for diagnosis, prediction of outcomes and the selection of high-risk individuals for SNVs.

### 3.7. Other Serum Biomarkers

A study concluded that neutrophil microparticles (NMPs) in MPO patients express higher levels of pentraxin-3 (PTX-3), high mobility group box 1 protein (HMGB1) and tumor necrosis factor-like weak inducer of apoptosis (TWEAK) when compared to HCs [48]. Higher levels of 12-Hydroxyeicosatetraenoic acid (12-HETE) in exhaled breath concentrates (EBCs) were reported in patients with EGPA when compared to asthma or hypereosinophilic syndromes [49]. Serum advanced glycation end products (sRAGE) were associated with inflammation in GPA. They thus could be used as disease activity biomarkers in mild or “limited” cases [50]. Therefore, although numerous studies have addressed novel biomarkers in primary SNV, few of these biomarkers are currently being used in routine clinical practice in the management of patients. Today, biomarker-driven treatment algorithms are unavailable in this type of vasculitis based on the insufficient utility and limitations of biomarkers for SNV.

## 4. Advances in Biomarker Research 

New tools for early disease detection, diagnosis, monitoring of therapeutic response and disease progression are needed to improve the mortality rate of patients with SNV and AAV, particularly. Recent discoveries and developments in biomarker research and identification in the early stages of SNV aim to increase the survival of these patients. Nevertheless, no single candidate could be used for all the roles as a single biomarker. Thus, integrating multimodal biomarker approaches will likely be necessary in the future. Using targeted serum proteomics, Ishizaki J et al. identified several circulating biomarkers for disease activity and predication of organ involvement in AAV. Along with TIMP-1 as the best disease activity biomarker, the authors also found tenascin C (TNC), CRP, leucine-rich alpha-2-glycoprotein 1, S100A8/A9, CD93, MMP-9, and transketolase (TKT) as biomarkers discriminating highly active AAV patients from those in remission. TKT and CD93 were first described as biomarkers for renal involvement and kidney outcome in AAV and TNC levels correlated with lung involvement in AVV patients [33]. 

Liu S et al. performed serum metabolic profiling of AAV patients with renal involvement and HCs and found that N-acetyl-L-leucine, Acetyl-DL-Valine, 5-hydroxyindole-3-acetic acid, and the combination of 1-methylhistidine and Asp-phe accurately distinguish patients from HCs. Moreover, 1-methylhistidine was significantly associated with the progression and prognosis of AAV with renal involvement [51]. 

Several tissue types are used for biomarker research in SNV and are shown in Table 1 [52,53,54,55,56].

A definitive diagnosis of necrotic vasculitis is based on the histological confirmation of necrosis in the vessel wall on the biopsy of an affected area. In cases where a biopsy is not recommended, the clinical context and the serum/plasma/urine biomarkers and/or imaging data are considered sufficient for the diagnosis. This includes the positivity of anti-PR3 ANCA in a high percentage of GPA, anti-MPO ANCA in around 60% of MPA and 1/3 of EGPA, or the presence of microaneurysms of the abdominal vessels on arteriography in PAN [57].

Considering that no single biomarker can be used to diagnose, predict disease activity or treatment outcome, the use of various imaging modalities can be particularly useful when assessing SNV patients. Some of the major imaging modalities used in the evaluation of such patients have been presented in Table 2.

However, although biomarkers for SNV are insufficient and have many limitations, the advent of emerging technologies sheds light on the landscape of biomarker identification. We can speculate that integrating multimodal approaches, a pioneering strategy that harnesses diverse technological platforms to enhance the depth and breadth of biomarker discovery, is the sole chance of advancing biomarker development. By combining genomics, proteomics, and metabolomics, researchers gain a holistic view of the molecular landscape associated with SNV, and new biomarkers can be identified and tested. Furthermore, genomic analysis explores genetic variations predisposing individuals to SNV, while proteomics elucidates protein expression patterns, unraveling critical players in the disease pathogenesis. Nevertheless, metabolomic profiling provides insights into the dynamic changes in metabolic pathways, offering valuable information on disease progression and response to therapy.

This integrated approach not only captures a more nuanced understanding of SNV but also overcomes the limitations of individual technologies, ensuring a more comprehensive biomarker panel. The synergy between these modalities holds the potential to identify novel, precise biomarkers with improved diagnostic accuracy and prognostic value. Moreover, advancements in computational biology and artificial intelligence contribute to the analysis of vast datasets, facilitating the identification of complex biomarker signatures. We can also conclude that integrating multimodal biomarker approaches represents a pivotal step toward precision medicine in SNV, offering a paradigm shift in diagnostic and prognostic strategies that can ultimately enhance patient outcomes.

## 5. Clinical Implications of Biomarkers for SNV 

### 5.1. Biomarkers for Diagnosis and Disease Subtyping and Prognostic Value of Biomarkers

Over the past few decades, a plethora of studies led to a better understanding of the pathogenesis of SNV. They revealed the potential role of many clinically relevant biomarkers for diagnosis, disease activity, relapsing rate, prognosis and treatment outcome. As emphasized above, SNV is a group of relapsing diseases, typically affecting small to medium vessels, with potential multiorgan involvement and characterized by necrotizing inflammation and endothelial injury [20]. At the beginning of our paper, we mentioned biomarkers associated with SNV and how they relate to one another. In the following paragraphs, we will focus on the clinical implications of these markers and the data supporting them. The immunodiagnostic approach in disease subtyping is based on ANCA serology and immune deposits in situ [58]. Since they were first reported in the early 1980s, ANCAs have been widely used as a diagnostic biomarker in patients suspected of necrotizing vasculitis [59].

Currently, only ANCAs have diagnostic potential for AAV in clinical practice. Although up to 85–95% of the patients with GPA, MPA, pauci-immune necrotizing and crescentic glomerulonephritis are ANCA-positive, autoantibodies correlate better with the disease phenotype rather than with the diagnosis. Up to 90% of the patients with active, generalized GPA are ANCA-positive compared to 40% of localized GPA. Moreover, around 90% of the antigen target is PR3 in the ANCA-positive GPA group. pANCAs are mostly associated with anti-MPO antibodies and are found in patients with small vessel vasculitis without evidence of granulomatous inflammation. Although the titer of ANCAs could fluctuate with disease activity, their predictive value for relapses is relatively low [60,61]. Data from the literature identify the serum persistence of ANCA and ANCA reappearance as risk factors for relapse, as well as the association of PR3-ANCA antibodies with more frequent relapse, higher risk for severe inflammatory lung disease, and systemic disease involving multiple organs at diagnosis compared to MPO-ANCA. The latter are associated with more severe renal limited involvement [62]. Patients with PR3-ANCA-positive AAV respond better to rituximab than to cyclophosphamide [7]. Interestingly, patients with lupus nephritis (LN) and positive ANCAs have more serologically active SLE and more necrotic areas on renal biopsy than ANCA-negative patients [63]. PR3-ANCA-positive samples have 99% specificity for GPA, and MPO-ANCA-positive samples have an 80% specificity for MPA [64]. The role of ANCAs as a diagnostic biomarker is indisputable, yet their predictive value for disease relapse and treatment response remains under investigation. Persistent ANCA positivity or rising titers, ANCA reappearance, and anti-PR3 antibodies are risk factors for relapsing disease, yet discordance between ANCA and disease activity is not unusual [65,66].

Gou et al. conducted an epitope analysis of anti-MPO in patients with AAV to evaluate the prognostic value of ANCAs. Patients with a recognizable N-terminus of MPO heavy chains were predisposed to more severe disease. In contrast, patients with low affinity would likely have lower vasculitic activity even though they may have higher serum levels of MPO-ANCA [67]. Studies have shown a better response to rituximab treatment in PR3-ANCA-positive patients in comparison to conventional therapy for induction and maintenance of remission with cyclophosphamide or azathioprine [20]. Hogan et al. investigated the correlation between lung involvement and the substantial predictive value of PR3-ANCA for disease relapse [61].

Novel antibodies have been recently described as possible candidates for diagnostic biomarkers. Anti-tissue plasminogen autoantibodies have been detected in up to 25% of anti-PR3 and anti-MPO-positive patients [65]. Berden et al. described their association with more severe glomerular inflammation and increased microthrombotic events [68]. Anti-LAMP2 autoantibodies are considered a subtype of ANCA and are described in up to 87% of patients with GPA, MPA and renal limited vasculitis (RLV). Kain et al. investigated their potential role in the pathogenesis of AAV and the predictive value for disease activity [69]. AECAs have been described in PAN, GPA, MPA, EGPA, IgAV and KD. However, their utilization as a diagnostic tool is yet to be determined [21].

Traditional inflammatory markers such as ESR or CRP, together with calprotectin, hepcidin and procalcitonin, are non-specific and only indicate inflammation without its exact etiology but still correlate with disease activity [16,17]. Pepper et al. investigated calprotectin as a potential disease biomarker in patients with AAV. They reported that patients with AAV have higher monocyte and neutrophil cell surface calprotectin expression than HCs and that its levels increased with treatment withdrawal and were significantly elevated in patients with disease flare-ups [18]. Currently, calprotectin is not used routinely in clinical practice as a biomarker for diagnosis or activity in AAV.

Some chemoattractant molecules such as eotaxin-3, immunoglobulin G4 (IgG4), and CC chemokine ligand 17 (CCL17/TARC) have been found at elevated levels in active EGPA compared to HCs and inactive EGPA and have a potential role as a diagnostic biomarker [70].

Rodriguez-Pla et al. examined 22 experimental serum proteins in different types of vasculitides. In PAN, ESR and MMP-3 showed significant changes during active disease regardless of treatment, and in EGPA, granulocyte colony-stimulating factor (G-CSF), granulocyte-macrophage colony-stimulating factor (GM-CSF) IL-6, IL-15, and sIL-2Rα showed significant increases during active disease, as did B cell-attracting chemokine (BCA)-1/CXCL13 but only after adjustment for treatment [71].

Another promising biomarker for diagnosis and subtype distinction is 12-Hydroxyeicosatetraenoic acid (12-HETE). In their study, Szczeklik et al. assessed the eicosanoid profile both in EBC and broncho-alveolar lavage fluid (BALF) of EGPA, hypereosinophilic syndrome (HES) and HCs. They identified higher exhaled breath concentrations of 12-HETE in EGPA patients compared to HCs and HES patients [49]. Circulating free DNA (cfDNA) is another disease subtyping biomarker. Lange et al. observed significantly increased serum levels of cfDNA in PR3-ANCA GPA patients compared to EGPA patients, and there was an association between the concentration of cfDNA and disease activity [44]. Urinary proteins are potential candidates for non-invasive biomarkers with prognostic value for renal involvement in AAV. Soluble CD163 (sCD163) has been investigated in the context of active renal vasculitis. Patients with small vessel vasculitis GPA, MPA and EGPA exhibit significantly higher urinary sCD163 levels than those in remission, disease controls or HCs [39]. Chen et al. explored neutrophil gelatinase-associated lipocalin (NGAL) as a relevant biomarker for the diagnosis and relapse of AAV. They found significantly higher levels of NGAL in active disease. NGAL has also been investigated as an early predictor of acute kidney injury (AKI) in AAV [72].

### 5.2. Monitoring Disease Activity and Treatment Response with New Biomarkers

While ANCAs participate in the pathogenesis of necrotizing vasculitides and have been used both to diagnose AAV and as markers of disease activity, they appear to not be specific or sensitive enough to address all disease and treatment-related outcomes [65,73,74]. Even though persistent ANCA positivity or ANCA reappearance after previous negativity, as well as high serum levels of PR3-ANCA, have all been associated with disease activity and risk of relapse, cases of seronegative disease, as well as ANCA positivity in the absence of disease, have somewhat limited its reliability [65,66]. In recent years, many novel biomarkers have been explored as indicators of disease activity and relapse or possible predictors of treatment response (Table 3). One such candidate is pentraxin-3 (PTX3), an acute-phase serum protein. A study on 79 subjects with newly diagnosed or relapsing AAV found significantly higher plasma and urine levels of PTX3 compared to HCs, which correlated with the Birmingham Vasculitis Activity Score (BVAS) at baseline, estimated glomerular filtration rate (eGFR) and degree of albuminuria [75]. In another study on a total of 101 patients with either GPA, EGPA or MPA, Padoan et al. reported that the presence of anti-PTX3 antibodies in the serum was associated with a lower prevalence of renal, ENT and systemic manifestations and was particularly useful as a biomarker in subjects with EGPA [76]. PTX3 may, therefore, be considered a marker of disease activity, especially in patients with renal involvement. NGAL is a protein involved in innate immunity and a marker of neutrophil degranulation. Circulating NGAL levels were higher in AAV patients at disease onset and relapse compared to remission. They were closely correlated with BVAS, CRP and ANCA [72]. Interestingly, NGAL appears to play a renoprotective role in AAV by downregulating Th-17 immunity and thus preventing the development of ANCA-mediated crescentic glomerulonephritis [77]. Therefore, higher serum levels of NGAL have been considered an early predictor of acute kidney injury in AAV [65]. MCP-1 and sCD163 are two urinary biomarkers that can be used together as indicators of a subtle renal flare [78]. In particular, urine levels of MCP-1 correlate with renal vasculitis activity and persistence and decrease after successful treatment [65,79]. Urinary sCD163 is strongly associated with more severe renal biopsy findings such as glomerular fibrinoid necrosis and formation of crescents both at vasculitis onset and relapse as opposed to patients in remission and HCs [80]. One longitudinal study of AAV patients examined an array of experimental markers, including cytokines, chemokines, acute phase reactants and tissue damage markers and found that CXCL13, IL-6, IL-8, IL-15, IL-18BP and MMP-3 were all positively and significantly correlated with disease activity. A rise in the levels of IL-8, IL-15 or IL-18BP was the most promising predictor of a future relapse in patients with prolonged remission [17]. Finally, while B cell count in peripheral blood has been routinely used to guide therapeutic decisions in patients on rituximab treatment, a recent trial explored the baseline expression of Fc receptor-like 5 (FCRL5), a marker of both naïve and memory B cells, as a biomarker of treatment response in patients with GPA and MPA [81]. The baseline level of FCRL5 was significantly higher in patients achieving and maintaining complete remission at 6, 12, and 18 months after rituximab treatment, which was not reported in the groups treated with cyclophosphamide and azathioprine [81].

## 6. Challenges and Future Directions

The utilization of biomarkers in SNV faces challenges related to standardization and validation. The heterogeneity of SNV, coupled with diverse patient populations, necessitates the establishment of standardized protocols for biomarker assessment. Achieving consensus on analytical methods, sample collection, and result interpretation is paramount for ensuring the reliability and reproducibility of biomarker data. Additionally, rigorous validation studies are essential to confirm the clinical utility of identified biomarkers, addressing issues of sensitivity, specificity, and robustness across various cohorts. Establishing standardized practices will enhance the comparability of results across studies and facilitate the translation of promising biomarkers from research settings to routine clinical use.

Nevertheless, successfully integrating biomarkers into clinical practice requires overcoming implementation barriers. Bridging the gap between research findings and real-world applications involves developing user-friendly assays compatible with routine clinical laboratories. Healthcare providers need guidance on interpreting biomarker results and their implications for diagnosis, prognosis, and treatment decisions. Furthermore, establishing clear clinical guidelines for incorporating biomarkers into existing diagnostic algorithms is crucial. Collaborative efforts between researchers, clinicians, and regulatory bodies are necessary to streamline this transition, ensuring that the benefits of biomarker-driven strategies reach patients promptly and effectively.

Finally, despite significant progress, unmet needs persist in SNV biomarker research, creating opportunities for further exploration. Deeper insights into the molecular mechanisms driving SNV pathogenesis could unveil novel biomarker candidates. Investigating the longitudinal dynamics of biomarkers in response to treatment and disease flares may enhance their predictive value. Exploring the potential of liquid biopsy and imaging modalities as complementary biomarker sources could provide a more comprehensive diagnostic and monitoring toolkit. Collaboration across multidisciplinary research teams and international consortia is imperative to address these unmet needs, fostering a holistic understanding of SNV and advancing the field toward innovative diagnostic and therapeutic strategies. Future research endeavors should prioritize these challenges, nurturing a continuous cycle of discovery, validation, and clinical implementation to benefit individuals affected by SNV.

## 7. Conclusions

In conclusion, the complexity of SNV demands a concerted effort to standardize biomarker evaluation, integrate them into clinical practice, and address ongoing research gaps. As we bridge these challenges, the promise of refined diagnostics and personalized treatment approaches emerges, offering hope for improved outcomes in SNV patients. The accomplishment of these hinges on collaborative endeavors, embracing technological advancements, and a relentless commitment to advancing our understanding of SNV for the improvement of patient care.

## Figures and Tables

**Table 1:** Types of biomarkers for SNV based on sample source and detection method [52,53,54,55,56].

Sample Source	Detection Method	Markers	References
Tissue	HE staining, phosphotungstic acid haematoxylin (PTAH) staining, elastic Masson staining, periodic acid methenamine silver–HE staining, Immunofluorescent staining for IgG and IgA, PAS staining, PAM-HE staining, EVG staining, immunohistochemistry, PCR-based techniques and microarray	Cellular crescents, neutrophil infiltration, erythrocyte extravasation, deposition of fibrin-like substances, necrotizing granulomas, fibronoid necrosis, IgG, IgA, necrosis; miRNA expression	[52,53]
Serum	Quantitative proteomics; ELISA	calreticulin, annexin-A1 and phospholipid scramblase 1 ANCA PR3-ANCA MPO-ANCA	[54]
Urine	Label-free LC-MS/MS mass spectrometry	Proteomes of small extracellular vesicles (EVs)—Golgi enzymes (MAN1A1)	[55,56]

Legend: ANCA—antineutrophil cytoplasmic antibodies; ELISA—enzyme-linked immunosorbent assay; EV—extracellular vesicle; EVG—Elastin van Gieson; HE—hematoxylin eosin; Ig—immunoglobulin; LC-MS/MS—liquid chromatography–mass spectrometry MAN1A1—Golgi α-1,2-mannosidase IA; miRNA—microribonucleic acid; MPO—myeloperoxidase; PAS—periodic acid–Schiff; PAM—periodic acid methenamine silver; PCR—polymerase chain reaction; PR3—proteinase 3; PTAH—phosphotungstic acid haematoxylin.

**Table 2:** Imaging modalities in the diagnosis of SNV.

Diagnostic Tool	Applications and Advantages	Limitations
Ultrasound	Visualization of vessels, including with Doppler modality	Poor visualization of some vessels Intersonographer variability
Computed tomography (CT)	Diagnosis and staging	Nephrotoxicity (Iodine-contrast agent); potential side effects from exposure to radiation
Magnetic resonance imaging (MRI)	Superior imaging Iodine-free contrast agent No radiation	Expensive Less availability
Positron emission tomography (PET)	Whole body scanning Monitoring recurrence and response to therapy	Expensive Less availability
Arteriography	Whole body scanning Monitoring recurrence and response to therapy	Nephrotoxicity (Iodine-contrast agent); potential side effects from exposure to radiation

**Table 3:** Novel biomarkers in assessing disease activity and treatment response in systemic necrotizing vasculitis.

Biomarker	Characteristics	Clinical Usefulness	References
Pentraxin-3 (PTX3)	Acute phase reactant	Disease activity monitoring, possible renal involvement	[75,76]
Neutrophil gelatinase-associated lipocalin (NGAL)	Marker of neutrophil degranulation	Disease activity monitoring, early predictor of acute kidney injury	[72,77]
Monocyte chemoattractant protein-1 (MCP-1)	Chemokine	Disease activity monitoring, renal flare	[78,79]
Soluble CD163 (sCD163)	Scavenger receptor for haemoglobin/haptoglobin complexes	Disease activity monitoring, renal flare; correlates with more severe biopsy findings	[78,80]
IL-8, IL-15, IL-18BP	Cytokines	Possible predictors of future relapse	[17]
Fc receptor-like 5 (FCRL5)	Marker of naïve and memory B cells	Possible predictor of response to rituximab therapy and sustained remission	[81]

## Data Availability

Data are contained within the article.
